# Advances in Methods for Accurate Prediction of RNA–Small Molecule Binding Sites: From Isolated to AI-Integrated Strategies

**DOI:** 10.3390/ph18101593

**Published:** 2025-10-21

**Authors:** Jiaming Gao, Chen Zhuo, Chengwei Zeng, Haoquan Liu, Yunjie Zhao

**Affiliations:** Institute of Biophysics and Department of Physics, Central China Normal University, Wuhan 430079, China

**Keywords:** RNA binding sites prediction, artificial intelligence, machine learning, deep learning

## Abstract

Predicting RNA–small molecule binding sites is essential for developing RNA-targeted drugs. Identifying these sites experimentally is often costly, making computational methods essential for drug discovery. While traditional approaches rely on limited information, recent AI advancements allow the integration of diverse features, improving prediction accuracy. As methods for predicting RNA–small molecule binding sites continue to evolve, this review provides a timely overview of recent developments. It systematically traces the evolution from physics-based, isolated strategies to AI-integrated approaches, explains the fundamental principles behind different features, compares the tendencies of various features between binding and non-binding sites, evaluates the performance of approaches using different feature combinations on various test sets, and outlines remaining opportunities and challenges, offering guidance for researchers aiming for higher prediction accuracy.

## 1. Introduction

One of the central tasks in drug discovery is to identify bioactive compounds that selectively bind to disease-relevant targets [1]. Achieving such selective binding requires precise localization of binding sites on the target molecule that can bind to small molecules. Over the past two decades, the development of computer-aided drug design has led to revolutionary advances in protein-targeted drug discovery [2,3]. Benefiting from the progress of artificial intelligence (AI) algorithms, the accuracy of identifying protein-small molecule binding sites has been continuously improved. However, protein-coding genes constitute only ~1.5% of the human genome, and merely 10–15% of these are considered druggable [4,5]. In contrast, more than 95% of the human genome is transcribed to non-coding RNA, which participates in diverse biological processes and offers a vast pool of untapped drug targets [6].

The characteristics of RNA sequences and structures are closely related to the druggability of RNA. RNA is a biomolecule with high sequence specificity. Different RNA sequences exhibit variations in both structure and function, which makes RNA a highly selective target for drugs. Additionally, the unique structure of RNA enables small molecules to bind to and interact with specific regions of RNA, thereby influencing its function. This sequence and structural precision are relevant not only to human cellular RNA but also to viral RNA. Numerous viruses possess RNA genomes, and small molecules that target RNA can suppress the transcription of RNA viruses, aiding in the prevention of pandemics caused by these viruses, including SARS-CoV-2 [7,8,9,10]. Therefore, RNA has attracted increasing research interest as a binding target for small molecules in recent years.

Accurate identification of small molecule binding sites in RNA facilitates the development of RNA-targeted therapeutics and advances the discovery of small-molecule drugs that selectively bind RNA. Small-molecule drugs targeting RNA have achieved breakthrough progress. For example, the synthetic small molecule Risdiplam functions as a splicing modifier of SMN2 pre-mRNA, regulating its alternative splicing and restoring the expression of functional SMN protein. Risdiplam has improved motor function in patients with spinal muscular atrophy, demonstrating that RNA can serve as a druggable target for small molecule [11,12,13]. Similarly, as a synthetic small molecule, Ribocil can selectively target the FMN riboswitch in bacteria. It mimics the function of the natural ligand FMN, blocking the transcription of downstream riboflavin biosynthesis genes. The success of Ribocil provided cellular proof that small molecule binding to a non-coding RNA structural element is sufficient to elicit antibacterial activity [14,15,16]. These examples collectively illustrate the druggable potential of RNA as a target for small-molecule therapeutics.

Nevertheless, experimentally screening small molecules that target RNA remains a challenging task. RNA molecules possess a highly charged backbone formed by phosphate groups and exhibit high flexibility and high polarity. The high flexibility of RNA structures can lead to unstable small molecule binding sites in high-throughput screening. In contrast, the high polarity affects binding specificity, increasing the likelihood of false-positive results. These structural characteristics make it challenging to experimentally determine the binding sites of RNA–small molecule complexes. In addition, experimentally determining the binding sites of RNA–small molecule is often time- and resource-consuming. Hence, developing highly accurate computational methods to recognize small molecule binding sites on RNA is vital for the discovery of RNA-targeted small-molecule drugs. With the increasing number of tertiary structures of RNA–small molecule complexes deposited in the Protein Data Bank (PDB), the development of high-precision computational algorithms has become increasingly feasible [17]. Hybrid strategies that combine experimental and predicted structures are also being employed to develop more accurate computational methods [18]. Moreover, RNA large language models (LLMs) effectively leverage RNA sequence data, which far exceeds the amount of RNA structural data. It can complement structure-based models, making it promising to improve the performance of computational methods further.

**Figure 1:**
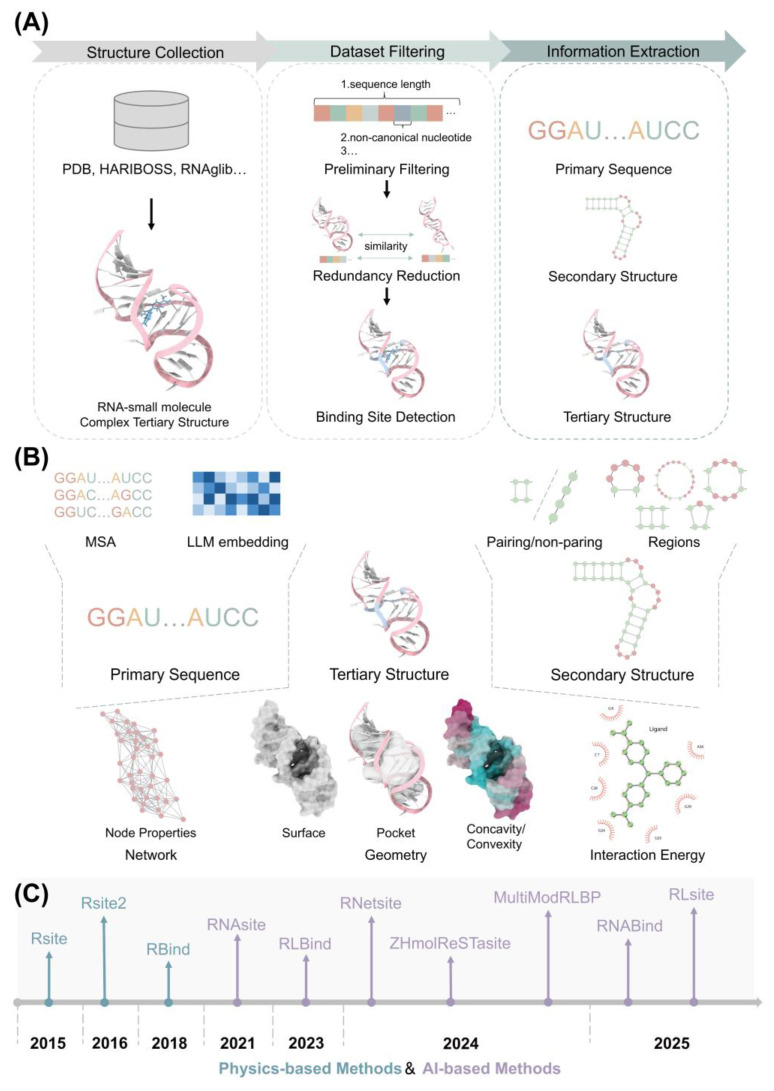
Overview of data preparation for RNA–small molecule binding site prediction. (**A**) Process of data collection and dataset construction including complex structure collection, dataset filtering and information extraction. (**B**) Feature extraction for predicting small molecule binding sites in RNA, incorporating features derived from RNA sequence, secondary structure and tertiary structure. (**C**) Timeline of methods for predicting RNA–small molecule binding sites, including both physics-based approaches and AI-based approaches.

Numerous computational methods have been developed to predict small molecule binding sites in RNA structures, as shown in Figure 1C. Early methods primarily characterized RNA nucleotides by extracting isolated physics-based information from RNA structures, relying on both RNA tertiary or secondary structures [19,20,21]. By coarse-graining the structures, these methods distinguished individual nucleotides within the structures to predict potential binding sites. Recently, driven by the breakthroughs of AI algorithms in structure prediction, many researchers have developed machine learning (ML) and deep learning (DL)-based methods that integrate diverse information to identify potential binding patterns of RNA–small molecule interactions [22,23,24,25,26,27,28,29,30]. Different from previous physics-based methods, most AI-driven approaches leverage information from multiple RNA modalities as complementary features, such as sequence and geometry [31,32].

This review offers a thorough and current overview of recent progress in predicting RNA–small molecule binding sites. We begin by outlining the main workflows of different methods and the feature types they utilize, then explain the fundamental principles behind these features. Next, we review how AI-based approaches construct datasets and assess their performance across various test sets, examining how the choice of features influences results. Finally, we discuss the main challenges and future opportunities in this field. Unlike earlier reviews mainly centered on RNA structure or physics-based methods, this review highlights AI-driven integrated strategies that utilize multimodal features, such as large language models, to predict RNA–small molecule binding sites.

## 2. Methods for Predicting RNA–Small Molecule Binding Sites

RNA–small molecule binding site prediction methods fall into two main categories: physics-based and AI-based. These approaches use different RNA data as inputs and combine various features. Table 1 details the input data, feature combinations, models, and available websites for each method. Below, the core workflows and features of these methods are explained.

### 2.1. Physics-Based Methods

*Rsite.* Rsite is a tertiary structure-based method to predict the functional sites of RNA [19]. Drawing inspiration from protein function prediction techniques, Rsite identified two key points: (1) Nucleotides with high closeness centrality in RNA tertiary structures tend to be functionally significant. (2) Surface-exposed nucleotides are more likely to engage in recognition and RNA interactions. Based on these insights, Rsite hypothesized that in non-coding RNA structures, both highly connected and less connected nucleotides are often functionally important, indicating potential functional sites.

Based on this hypothesis, Rsite recognize functional sites as following steps: (1) for a given RNA tertiary structure with n nucleotides, Rsite calculates the Euclidean distance between every pair of nucleotides, yielding n×(n−1) distance values. (2) for each nucleotide, Rsite calculates the sum distances to the other (n-1) nucleotides. For an RNA tertiary structure with n nucleotides, generate a distance curve comprising n data points. (3) a Gaussian filter is applied to smooth the distance values for each nucleotide. Specifically, Rsite applies a sliding window of size w to each nucleotide, computing the average distance values within the window:

(1) $$S_{i}=\frac{D_{i-w}+\cdots +D_{i}+\cdots +D_{i-w}}{2w+1}$$

where $D_{i}$ denotes the sum of distances between i-th nucleotide and all other nucleotides, w denotes the window size and $S_{i}$ corresponds to the smoothed distance value for i-th nucleotide. Rsite implemented a window size of 2 in their algorithms. (4) on the smoothed distance curve, local minima and maxima are identified as putative RNA functional sites. In addition, both start and end points are considered functional sites if their distances deviate by more than 50% from the mean distance.

*Rsite2.* RNA’s secondary structure is simpler to obtain than its tertiary structure, which makes analyzing RNA functional sites based on secondary structure more efficient. Building on this concept, Zeng et al. [20] examined RNA secondary structure and discovered that, in non-coding RNAs, the distance from a nucleotide to the centroid in the secondary structure is strongly positively correlated with its distance to the centroid in the tertiary structure. This indicates the potential for predicting functional sites using distance metrics derived from secondary structure. Based on this, Zeng et al. introduced Rsite2, a computational method for identifying RNA functional sites from secondary structure. Rsite2 takes an RNA sequence as input, predicts its secondary structure with RNAfold, and extracts the 2D coordinates of each nucleotide from the RNAfold output [33]. Rsite2 introduces two metrics to illustrate the position of each nucleotide in the RNA: the nucleotide-to-centroid distance, indicating the Euclidean distance between a nucleotide and the centroid, and the nucleotide distance sum, which totals the distances from that nucleotide to all others. Rsite2 computes these two metrics and uses the same algorithm as Rsite to predict RNA functional sites: for an RNA composed of *n* nucleotides, a distance curve of length *n* is plotted, where position *i* represents the *i*-th nucleotide. The curve is smoothed using a Gaussian filter, and nucleotides located at the extrema of the curve are identified as functional sites.

*RBind.* Wang et al. observed that while Rsite and Rsite2 can predict functional sites solely based on distances from RNA secondary and tertiary structures, they may generate false positives because they do not differentiate between strong and reliable connections. To improve this, Wang et al. created RBind, a computational approach that converts RNA tertiary structures into network representations [21]. Given an RNA tertiary structure, RBind treats nucleotides as nodes and their non-covalent interactions as edges to build an undirected network. Each nucleotide is characterized by network properties. An edge is added between two non-adjacent nucleotides if any pair of their heavy atoms is within 8 Å. RBind then calculates two network metrics, degree and closeness, to identify functional sites. A nucleotide is considered a functional site if both its degree and closeness are more than one standard deviation above the average values for all nodes.

### 2.2. AI-Based Methods

*RNAsite.* RNAsite is a machine learning method based on RNA sequences and tertiary structures, composed of two modules: sequence-based RNAsite_seq and tertiary structure-based RNAsite_str [22]. RNAsite_seq utilizes RNA sequences as input, performs BLASTN searches against the NCBI nucleotide database to construct multiple sequence alignments (MSA) [32]. The results of MSA are then employed to compute evolutionary conservation scores for each position in the RNA sequence. For each nucleotide, RNAsite_seq considers a window size of 12 to take its sequence context neighbors from a total of (2×12+1) nucleotides into account, then uses a Random Forest algorithm to implement training and predicting processes. RNAsite_str utilizes RNA tertiary structure as input, calculates four structure-based features: accessible surface areas (ASA), Laplacian norm (LN), degree, and closeness [34,35]. Similarly to RNAsite_seq, RNAsite_str uses a window size of 14 to capture the context neighbors of target nucleotides, and then the structure-based features of each nucleotide are sent to a second Random Forest model. Additionally, using a window size of 16, the prediction results from RNAsite_seq were integrated with structure-based features, and the combined features were used to train the final Random Forest model in RNAsite. RNAsite combined RNA sequence and structural information, outperformed the sequence-only module RNAsite_seq and the structure-only module RNAsite_str.

*RNetsite.* Liu et al. proposed RNetsite, a machine learning method for predicting small molecule binding sites by modeling RNA tertiary structures as networks [24]. RNetsite converts RNA tertiary structures into networks, with nucleotides as nodes and non-covalent interactions as edges. An undirected edge connects two nucleotides if any pair of their heavy atoms are within 8 Å. RNetsite uses network analysis and machine learning to predict small molecule binding sites. The process involves calculating network properties for each nucleotide, including two local features (degree and neighborhood connectivity) and three global features (closeness, betweenness centrality, and eccentricity). To capture the influence of surrounding nucleotides, RNetsite employs a sliding window of size w, encoding each nucleotide’s features by combining network properties from its 2w + 1 neighborhood. These features are then used to train three machine learning models: Random Forest (RF), Light Gradient Boosting Machine (LGBM), and Extreme Gradient Boosting (XGB). Predictions from the three models are combined using a voting strategy, where the final prediction for each nucleotide is made when at least two models agree.

*RLBind.* RLBind is a deep learning method that utilizes convolutional neural networks to extract both global and local information from RNA sequences and tertiary structures, capturing long-range and short-range interactions, respectively [23]. The method integrated nucleotide types and evolutionary conservations as sequence-based features, with the evolutionary conservations collected by multiple sequence alignment information of RNA sequences, acquired through BLASTN, into ConSurf-DB [32,36,37]. From RNA tertiary structures, two network features (degree, closeness), accessible surface areas and two biochemical properties (molecular mass, side-chain pka) were derived as structural features. RLBind employed a dual-channel architecture: a local channel that used an 11-nucleotide sliding window to capture the context of each nucleotide, and a global channel that processed fixed length (64-nt) RNA sequences. Features from the global channel were initially processed through a convolutional layer, then combined with those from the local channel, and finally passed into two dense layers. The RLBind prediction was produced by applying a sigmoid activation function to the output of these dense layers.

*ZHmolReSTasite.* Gao et al. developed a deep learning method, ZHmolReSTasite (ZeSTa for short), using RNA surface topography strategy to predict RNA–small molecule binding sites [25]. For an RNA tertiary structure, ZeSTa employed RNA surface topography as follows: the first step was to calculate the solvent-excluded surface and convert it into a cloud of vertices with 3D coordinates. Coordinates conversion was then applied to each nucleotide by rotating the RNA surface to align the geometric centers of nucleotides with the intersection of the equator and the prime meridian of the RNA surface. Three-dimensional coordinates of vertices were transferred to longitude and latitude coordinates, and vertices located within a 12Å geodesic radius from each nucleotide’s geometric center were extracted to represent the nucleotide. An interpolation method is then utilized to obtain regular topography images of nucleotides. The topography images were characterized based on RNA sequences, secondary structures, and tertiary structures. ZeSTa obtained multiple sequence alignment (MSA) for each RNA sequence by BLASTN searches against the NCBI nucleotide database. The results of MSA were utilized by the plmDCA method to analyze the coevolutionary relationship between nucleotides [32,38,39]. Secondary structure type information was derived from tertiary structures using RNAstat [40]. The tertiary structure features utilized by ZeSTa included three geometric-based features (SASA, LN, and pocket) and two network-based features (degree and closeness), as well as pKa values, which were incorporated based on nucleotide types. These features were added to the corresponding vertices of each nucleotide in the topography image, generating feature maps of nucleotides. ZeSTa then fed the nucleotide feature maps into a deep learning algorithm, ResNet18, for prediction in the RNA–small molecule binding site task [41].

*MultiModRLBP.* MultiModRLBP is a multimodal deep learning framework developed by Wang et al. [26]. The core concept involves encoding nucleotides through three perspectives: RNA structure relationship graph, nucleotide structural properties, and sequence semantics. Specifically, the method utilizes x3dna-dssr to extract two types of information from RNA tertiary structures: DSSR adjacency annotation and DSSR properties [42]. The DSSR adjacency annotation describes nucleotide neighborhoods based on the Leontis-Westhof system, where each nucleotide serves as a node, and interactions are categorized into 20 types of RNA base pairs. Network metrics like degree and closeness are derived from this annotation. DSSR also provides structural details such as torsion angles and key atomic coordinates. MultiModRLBP integrates these networks and DSSR properties with three types of accessible surface area and base encoding, resulting in 71-dimensional features per nucleotide. The process involves three components: (1) RNA graph. RGCN learns the RNA structural graph, with nodes representing nucleotides with 71D features and edges indicating Leontis-Westhof base interactions. (2) Nucleotide. The ELBFS module combines local features from a sliding window, global features from CNN, and individual 71D features to produce ELBFS features. (3) RNA semantic sequence. RNABERT, a pre-trained LLM for RNA sequences, is utilized to generate context-sensitive distributed word representations for each nucleotide [43]. Features from these three components are concatenated and fed into dense layers, producing prediction results of MultiModRLBP.

*RNABind.* Building upon the remarkable advances of LLMs in protein and DNA research, Zhu et al. [27] developed RNABind, a deep learning framework that integrates RNA LLMs with graph representations of RNA tertiary structures. Unlike the above methods, RNABind exclusively utilizes vector representations of nucleotides derived from RNA LLMs to characterize the nucleotide context. These context vector representations are used as node features in a network based on RNA tertiary structure. In this network, nodes are defined as the centroid of each nucleotide, with coordinates defined as the average of all atomic coordinates of the nucleotide. Nodes are connected to their 15 nearest neighbors via edges using a Euclidean distance-based k-NN strategy, with edge features consisting of distance encoded by 16 radial basis functions (RBFs). RNABind inputs node features, edge features, and nucleotide centroid coordinates into E(3)-equivariant graph neural networks (EGNNs) [44]. It processes the RNA graph representations through a stack of three equivariant graph convolutional layers (EGCLs), followed by a predictor that performs per-nucleotide classification to generate RNABind’s predictions. RNABind employs eight single-nucleotide resolution RNA LLMs and compares their performance in predicting RNA–small molecule binding sites [43,45,46,47,48,49,50,51]. The LLMs ERNIE-RNA and RiNALMo demonstrated superior performance across multiple evaluation metrics and were therefore selected as the primary models for reporting in RNABind.

*RLsite.* Zou et al. suggested that interaction energies are crucial for identifying nucleotides involved in small-molecule binding, including π-π stacking interactions, electrostatic interactions, and hydrogen-bond interactions [28]. To achieve this, they introduced RLSite, a deep learning method that integrates nucleotide interaction energy with structural and sequence information. RLSite uses a dual-channel architecture to process different feature types separately. The first channel converts the RNA’s tertiary structure into a 3D voxel grid, performs probe scanning to identify potential binding regions, and calculates interaction energies between RNA and 18 types of atomic probes using the ITScore-NL pretrained statistical potential [52]. For each probe type, the top 10 grid points with the most favorable binding energies are selected. The distances from each nucleotide’s six-membered ring center to these grid points are computed, and the energy values are mapped to nucleotides via inverse-distance weighting, forming a 10×18 energy feature map. A spatial sliding window is applied to stack the target nucleotide and its neighboring nucleotides, producing a (2w+1)×10×18 (with w=5) energy tensor. This tensor is processed by a 3D-CNN and a self-attention mechanism along the probe dimension to produce weighted spatial energy features [53].

The second channel integrates structural and sequence descriptors, including network properties (degree and closeness), Laplacian Norm, solvent-accessible surface area, and evolutionary conservation score, which is computed by plmDCA. These features are similarly aggregated with a sliding window of w=5 and fed into a BiLSTM to capture bidirectional contextual dependencies among nucleotides. Finally, the outputs from both channels are concatenated and passed through a fully connected layer to generate the prediction result of RLSite.

## 3. Feature Construction

RNA information can be classified into three categories: sequence, secondary structure, and tertiary structure, as shown in Figure 1B. Various features can be derived from these three types of information. The features extracted from these categories for predicting RNA–small molecule binding sites are summarized in Table 2, including the RNA information type, brief principle, available websites, and related methods. The fundamental principles of each feature are explained below.

### 3.1. Sequence-Based Features

*Multiple Sequence Alignment.* Multiple sequence alignment (MSA) is a technique that searches for homologous sequences of a target sequence in a database and then aligns the homologous or functionally related segments [25]. The MSA of RNA sequences reveals similarity and evolutionary relationships between sequences and has been successfully applied to RNA-related tasks. Evolutionary information extracted from MSA has been shown to improve the performance of RNA structure prediction significantly. Compared to relying solely on a single sequence, the incorporation of MSA can provide evolutionary information of nucleotides, such as evolutionary conservation. In predicting RNA–small molecule binding sites, the evolutionary information of nucleotides introduced by MSA can provide valuable additional information for characterizing small molecule binding sites. The MSA of RNA can be acquired from the BLASTN Field, which searches against the nucleic acid FASTA database provided by BLAST, and outputs the target RNA sequence along with its homologous sequences [32]. RNAsite, RLBind, ZeSTa, and RLsite all utilize MSA to derive evolutionary insights from RNA sequences [22,23,25,28]. RNAsite directly employs the MSA results to compute conservation scores, while the other three methods perform additional processing with direct coupling analysis.

*Direct Coupling Analysis.* The homologous sequences derived from the MSA contain information about RNA sequence conservation. Performing direct coupling analysis (DCA) on these sequences can uncover co-evolutionary relationships between nucleotides. Such relationships refer to interdependent changes happening at two nucleotides over evolutionary time, where a mutation in one often prompts a change in the other to maintain structural or functional stability. When analyzing these relationships in MSA, mutual information can be split into direct and indirect interactions. Direct interactions reflect actual correlations, while indirect interactions are mediated through other nucleotides. Relying only on mutual information makes it hard to distinguish between these types. DCA uses a statistical model to effectively differentiate direct from indirect interactions, emphasizing the direct nucleotide interactions usually linked to structural and functional roles. RLBind, ZeSTa, and RLsite use the plmDCA algorithm to derive evolutionary constraint information between nucleotides. Specifically, RLBind obtains plmDCA results via the online software ConSurf-DB, while ZeSTa and RLsite generate plmDCA outputs through local programs.

*Large Language Model*. Large language models (LLMs) were originally developed in the field of natural language processing and were later applied to the study of biomolecules. Since ESMfold achieved success in protein structure prediction with biological LLMs, RNA LLMs have also been rapidly developed and applied to many tasks, such as RNA structure prediction and small molecule binding site prediction [54]. RNA LLMs typically consists of two phases: pre-training and fine-tuning. During the pre-training phase, models are trained using RNA sequences from RNA sequence databases. The amount of RNA sequence data used for pre-training is usually enormous; for example, RNA-FM was pretrained on 23 million ncRNA sequences from the RNAcentral database [51,55]. Pre-training on extensive RNA sequence data allows the model to learn about sequence patterns and contextual meanings, helping it to grasp the fundamental functional and structural details. Afterward, for a specific downstream task, the model can be fine-tuned with task-specific datasets or used to produce a fixed-dimensional embedding for each nucleotide in an RNA sequence. These embeddings act as contextual-semantic features that highlight the sequential properties of nucleotides. MultiModRLBP and RNABind leverage RNA LLMs to encode nucleotides from RNA sequences. Specifically, MultiModRLBP uses RNABERT, while RNABind assesses and compares eight RNA LLMs, including RNABERT and RNA-FM, to determine their effectiveness in predicting RNA–small molecule binding sites.

### 3.2. Secondary Structure-Based Features

RNA folding largely depends on its base-paired structure, known as the RNA secondary structure. This secondary structure is essential for the proper function of many RNA molecules. In interactions between RNA and small molecules, binding sites are frequently found near the loop regions of the secondary structure. This pattern indicates that using secondary structure information to analyze nucleotides could improve predictions of RNA–small molecule binding sites.

There are two methods for representing RNA secondary structure: dot-bracket notation and region notation. Dot-bracket notation is a concise way to depict RNA secondary structure, showing how nucleotides are paired within an RNA molecule. In this notation, dots and parentheses are used; a dot ‘.’ indicates the nucleotide at that position is unpaired, while left/right parentheses indicate that the nucleotide is paired with a subsequent or preceding nucleotide. This method clearly illustrates which nucleotides are paired and which are not. The ZeSTa method, mentioned above, characterizes nucleotides based on the secondary structure regions they occupy, such as stems, internal loops, bulge loops, hairpin loops, and junction loops. Additionally, ZeSTa compared the performance of dot-bracket notation and region notation for characterizing RNA secondary structure and found that region notation, which offers a more detailed classification of the secondary structure regions of nucleotides, is more effective than simply classifying nucleotides as paired or unpaired using dot-bracket notation.

### 3.3. Network-Based Features

For an RNA consisting of n nucleotides, its structure can be represented as a nucleotide network, where nodes correspond to individual nucleotides and edges indicate non-covalent interactions between them. Network properties can then be used to characterize each node within this network. These properties, used in predicting RNA–small molecule binding sites, are classified into local and global categories based on their attributes. Local properties include degree and neighborhood connectivity, whereas global properties encompass closeness, betweenness centrality, and eccentricity. The following section details the definitions and equations for these network properties.

*Degree.* Degree centrality quantifies the number of edges connected to a node x, indicating its local connectivity. In nucleotide networks, degree reflects the direct interactions between a specific nucleotide node and its neighbors. Nodes with higher degree values show greater local connectivity and often act as binding sites for small molecules.

*Neighborhood Connectivity*. Neighborhood connectivity refers to the average degree of all the neighbors of a node x, characterizing the average number of connections of the node’s neighbors. Nodes with high neighborhood connectivity are often located in densely connected core regions of the network, and are more likely to serve as binding sites of small molecules. The neighborhood connectivity (NC) of node x is defined as:

(2) $$NC\left(x\right)=\frac{\sum DG\left(y\right)}{DG\left(x\right)}$$

where DG denotes the degree value and y is the neighbor of x.

*Closeness centrality*. A node’s closeness centrality is the inverse of the sum of its shortest path lengths to all other nodes, measuring the average distance from the node to the others. A higher closeness value indicates that the node is closer to the other nodes and is located nearer to the geometric center of the network, making it more important within the network. In nucleotide networks, binding sites often have higher closeness values. The closeness centrality of a node x can be calculated as follows:

(3) $$C\left(x\right)=\frac{n-1}{\sum_{x\neq y}d(x,y)}$$

where d(x,y) is the shortest path distance between node x and any other node y, n is the number of nodes in the network.

*Betweenness centrality*. Betweenness centrality measures how often a node appears on all shortest paths in a network. Nodes with higher betweenness values lie on more shortest paths and play a more important role in the flow of information within the network. In interactions with small molecules, nucleotide nodes that are more crucial in information flow are more likely to serve as binding sites. Betweenness centrality (BC) of a node x can be calculated as follows:

(4) $$BC\left(x\right)=\sum_{m\neq x\neq n}\frac{\sigma_{mn}(x)}{\sigma_{mn}}$$

where $\sigma_{mn}$ is the number of shortest paths from node m to node n, and $\sigma_{mn}(x)$ is the number of shortest paths from node m to node n that pass through node x.

*Eccentricity*. Eccentricity is defined as the number of steps required from node x to reach any other node in the network. It measures the maximum distance from node x to all other nodes in the network. Nodes with high eccentricity are far from the most distant nodes and are located near the network’s periphery, whereas nodes with low eccentricity are closer to the most distant nodes and are located near the network’s center. In nucleotide networks, nucleotides that bind to small molecules often have lower eccentricity. The calculation of eccentricity (EC) for node x is given by:

(5) $$EC\left(x\right)=\underset{x\neq y}{max}\;d(x,y)$$

where d(x,y) denotes the distance between node x and y.

### 3.4. Geometry-Based Features

*Solvent Accessible Surface Area.* The Solvent Accessible Surface Area (SASA) is defined as the surface area traced by the center of a spherical probe, roughly the size of a water molecule, as it rolls over the RNA surface. It indicates how much of the RNA is accessible to solvent molecules, thus reflecting the exposure level of different RNA regions [56]. A nucleotide with a higher SASA is more exposed, while one with a lower SASA is more buried within the RNA structure. Among the methods listed, RNAsite, RLBind, ZeSTa, MultiModRLBP, and RLsite utilize SASA to describe nucleotide properties. Overall, SASA is a key feature for characterizing the structural properties of nucleotides.

*Laplacian Norm*. The surface features of biomolecules are vital for understanding their properties and function as a key geometric indicator [31]. Laplacian Norm (LN) quantifies the concavity and convexity of nucleotides. Nucleotides with higher LN values have a more convex surface, while those with lower values are more concave. LN reflects the geometric features of nucleotides and is derived by measuring the distance between a target nucleotide and the weighted centroid of neighboring nucleotides. The process involves two steps: first, applying the Laplacian operator, and second, calculating LN. The Laplacian operator is defined as:

(6) $$\Omega_{ij}\left(\sigma \right)=\left\{\begin{array}{c} e^{-\frac{{\left\|p_{i}-p_{j}\right\|}^{2}}{\sigma^{2}}},\;\;if\;\left|i-j\right|>1\\ 0,\;\;otherwise \end{array}\right.$$

where $p_{i}$ and $p_{j}$ denote the C3 atom coordinates of nucleotide *i* and *j*, respectively. σ is a tunable parameter that acts as a scale factor. A smaller value of σ makes the Laplacian operator more sensitive to adjacent nucleotides, while a higher value of σ incorporates contributions from more distant nucleotides. Once the Laplacian operator is obtained, LN can be computed as:

(7) $${LN}_{i}\left(\sigma \right)=\left\|p_{i}-\frac{\sum_{j}^{\left|i-j\right|>1}\left[p_{i}\times \Omega_{ij}\left(\sigma \right)\right]}{\sum_{j}^{\left|i-j\right|>1}\Omega_{ij}\left(\sigma \right)}\right\|$$

Several approaches, including RNAsite, ZeSTa and RLSite, have employed LN as a feature to characterize nucleotides. Specifically, these approaches adopt multiple σ values (0, 1/4, 1/2, 3/4, 1) to consider the influence of surrounding nucleotides across different scales, thereby characterizing nucleotide concavity and convexity across scales ranging from local to global.

*Pocket*. The RNA pocket region is the internal cavity formed by nucleotides in RNA tertiary structures. Small molecules tend to bind to pocket regions when interacting with RNA molecules [57,58,59]. Therefore, compared to non-pocket nucleotides, pocket nucleotides are more likely to be small molecule binding sites. By classifying a nucleotide as a pocket/non-pocket type, properties of the nucleotide can be distinguished from a geometric perspective. Many approaches for detecting RNA pockets have been developed, such as 3V, MSPocket, PocketFinder, CHUNNEL, Ghecom [60,61,62,63,64,65,66]. These approaches use different methodologies to detect RNA pockets, and the detected pockets show variations. In addition, different RNA pockets often have varying sizes and depths, and these differences can affect the pocket nucleotides’ potential to bind small molecules. Using solely binary labels to mark nucleotides as pocket or non-pocket type, the geometric information of the pockets formed by nucleotides will be lost. The Ghecom method provides a measure, pocketness, to quantify the size and depth of the pocket formed by each nucleotide. This quantification of the geometric characteristics of pockets formed by different nucleotides can further subdivide pocket nucleotides. On this basis, ZeSTa utilizes probes of various sizes with the Ghecom algorithm to determine the pocketness of nucleotides when detecting pockets at different scales, thereby providing a comprehensive description of the geometric properties of nucleotides in forming pockets.

### 3.5. Energy-Based Features

The characterization of nucleotide energy properties explains the physical forces involved in RNA–small molecule interactions. These forces support the structural stability of RNA-ligand complexes and include long-range interactions, electrostatic forces, and four types of short-range interactions: stacking, hydrogen bonding, van der Waals forces, and hydrophobic interactions. Since RNA carries a strong negative charge, electrostatic interactions are especially crucial for RNA-ligand binding, as they facilitate long-distance recognition between RNAs and ligands [9,67]. In contrast, short-range interactions are associated with the optimization and stabilization of the complexes. For example, as one of the main features in nucleic acid structures, stackings are the non-covalent attractive interactions between aromatic rings and are crucial for stabilizing nucleic acid structures [28]. Hydrogen bonds are interactions that occur between a hydrogen atom and two electronegative atoms, like oxygen and nitrogen. The van der Waals interactions are weaker forces resulting from temporary uneven charge distributions between atoms [68,69]. Hydrophobic interactions are driven by the increase in entropy of surrounding water molecules when hydrophobic groups aggregate [70,71]. To consider these energy-related interactions, RLsite employed the potential function ITScore-NL to include nucleotide interaction energy features. ITScore-NL is a potential-based scoring system with three components: pairwise potential, stacking potential, and electrostatic potential.

## 4. Datasets for Predicting RNA–Small Molecule Binding Site: Training and Evaluating

Developing AI-based methods requires a training set, while evaluating their performance needs test sets that do not overlap with the training data [18]. Consequently, the process of constructing datasets is vital for the development of methods to predict RNA–small molecule binding sites. Figure 1A shows the three steps involved: structure collection, dataset filtering, and information extraction. Since known binding data for RNA–small molecule complexes is essential, data collection typically begins with gathering resolved structures of RNA–small molecule complexes from tertiary structure databases, such as PDB, HARIBOSS, and RNAglib [17,72,73]. Subsequently, dataset filtering is performed to eliminate redundancy. Initial filtering typically removes structures that do not meet criteria such as excessively long or short RNA sequences or the presence of non-canonical nucleotides. Redundancy is then further reduced based on sequence and structural similarity, aiming to exclude highly similar structures. Finally, nucleotides in RNA that interact with small molecules are designated as binding sites, using a 4 Å cutoff: if any atom of a nucleotide is within 4 Å of any atom of the small molecule, it is considered a binding site. These labeled structures are then used for feature extraction, including sequence, secondary, and tertiary structures, to facilitate training and testing.

### 4.1. Training Sets

#### 4.1.1. TR60

The TR60 dataset, derived from the RNAsite methodology, has been employed to train models such as RNAsite, RNetsite, RLBind, ZeSTa, RNABind, and RLsite. Su et al. initially collected 712 RNA–small molecule complexes (excluding water molecules), with RNA lengths ranging from 20 to 1500 nucleotides [22]. Structural similarity between RNA structures was assessed using TM-scoreRNA, and a cutoff of 0.3 was applied to eliminate redundant structures: if the TM-scoreRNA between two structures exceeded 0.3, the structure with a higher proportion of binding sites was kept. After removing redundancies, 78 RNA structures remained. These were then clustered into 57 groups at a 30% sequence similarity cutoff. From these, 60 RNAs in 42 groups formed the training set TR60, while 18 RNAs in 15 groups constituted the testing set TE18.

#### 4.1.2. TrainRLBP

Dataset TrainRLBP is the training set for the method MultiModRLBP. Wang et al. extracted 810 RNAs with small molecule binding information (comprising 1248 RNA chains) from datasets provided by the Python package RNAglib [26,73]. Due to the length limitations of the LLM RNABERT used in MultiModRLBP, RNA chains longer than 440 nt were excluded. RNA chains with fewer than 4 binding sites were also excluded, resulting in a final set of 653 RNA chains, which, along with 228 small molecules, formed 1012 RNA–small molecule complexes. Wang et al. combined these 653 RNA chains with TR60, resulting in a total of 666 RNA chains. MMseq2 was used to calculate the sequence similarity among the RNA chains, and the 666 chains were clustered into 180 groups at a similarity cutoff of 30%. After removing 20 groups included in the test sets, the remaining 160 groups contained 561 RNA chains. These chains formed 851 complexes with 196 small molecules and were designated as the TrainRLBP training set for MultiModRLBP.

#### 4.1.3. RNABind Training Set

When compared to other methods, RNABind was trained on the TR60 dataset. However, Zhu et al. noted that TR60 includes only single-chain RNA structures. They aimed to focus on the entire RNA complex structures as a single unit, rather than on individual chains [27]. To achieve this, they built a new dataset for predicting small molecule binding sites. The process involved first extracting 862 RNA–small molecule complexes from the HARIBOSS dataset (as of 18 September 2024). Zhu et al. then excluded structures with RNA sequences longer than 500 nucleotides, as such structures display a significant imbalance between binding and non-binding sites and are predominantly RNA-protein complexes. Structures containing modified nucleotides were also removed. In the end, 353 RNA structures remained for training and testing the RNABind model.

### 4.2. Evaluation

#### 4.2.1. Test Sets

**Figure 2:**
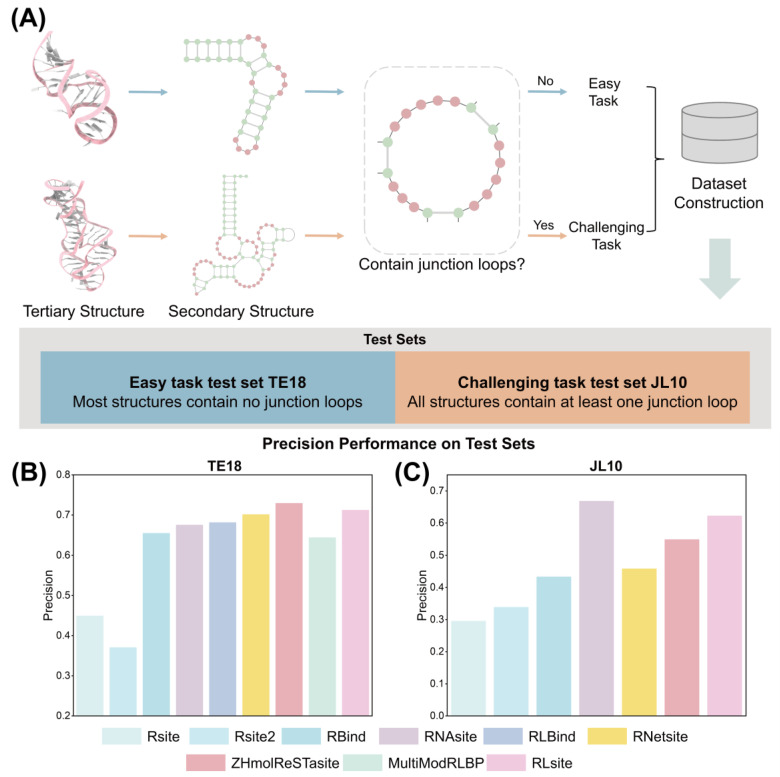
Division of test sets and precision performance of methods. (**A**) RNA structures are divided into easy and challenging prediction tasks based on whether junction loops are in their secondary structures. The easy task test set TE18 and challenging task test set JL10 are constructed under this criterion. (**B**) The precision performance of methods on test set TE18. (**C**) The precision performance of methods on test set JL10.

According to the ZeSTa method, predicting RNA–small molecule binding sites is categorized into easy and challenging tasks based on the presence of junction loops in the RNA secondary structure, as illustrated in Figure 2A. Easy tasks involve RNAs without junction loops, resulting in relatively simple structures. Conversely, RNAs with junction loops exhibit increased structural complexity, rendering the prediction of binding sites more challenging. Therefore, the evaluation of methods for predicting RNA–small molecule binding sites is divided into assessments of both easy and challenging tasks.

During the construction of the training set TR60, 60 of the 78 RNAs were selected for training, while the remaining 18 RNAs formed the test set TE18. Most structures in TE18 lack junction loops in their secondary structures, making it a simple test set for evaluating binding site prediction methods. Gao et al. constructed a more challenging, task-specific test set called JL10 [25]. Specifically, all RNA structures released after January 2021 from the PDB were included, provided they do not interact with proteins or DNA and have a resolution better than 4Å, resulting in 263 complexes. RNA chains that did not interact with small molecules and lacked junction loops in their secondary structures were then removed, leaving 76 RNA chains. Further, RNAs with more than two junction loops or binding sites making up less than 10% of the structure were excluded. To prevent redundancy, cd-hit was used for clustering, yielding 10 unique RNA structures [74]. All these RNAs contain at least one junction loop and constitute the challenging task test set JL10. We used JL10 to evaluate the performance of the methods on difficult cases.

#### 4.2.2. Feature Tendency in Easy and Challenging Tasks

To investigate the influence of different features on binding site prediction, we computed nucleotide features in the TE18 and JL10 test sets and analyzed the tendency between binding and non-binding sites. Specifically, we calculated the feature value for each nucleotide. We performed normalization within each RNA chain (for SASA, normalization was conducted within nucleotides of the same type to obtain the relative SASA). Then, the nucleotides in the datasets were ranked according to their normalized feature values in descending order. The ranked nucleotides were divided into 10 groups. We calculated the ratio of binding sites to the total number of nucleotides in the two test sets, which was 0.32 for both TE18 (195/605) and JL10 (265/827). Therefore, for the 10 groups of nucleotides after ranking, if the proportion of binding sites within a group exceed 0.32, we regarded it as a binding-site tendency group and filled it in green; otherwise, it was considered a non-binding-site tendency group (5 nucleotides in TE18 and 7 nucleotides in JL10 were placed in additional groups due to the total number of nucleotides not being divisible by ten). Subsequently, we were able to obtain the feature tendencies of binding sites from the figures.

**Figure 3:**
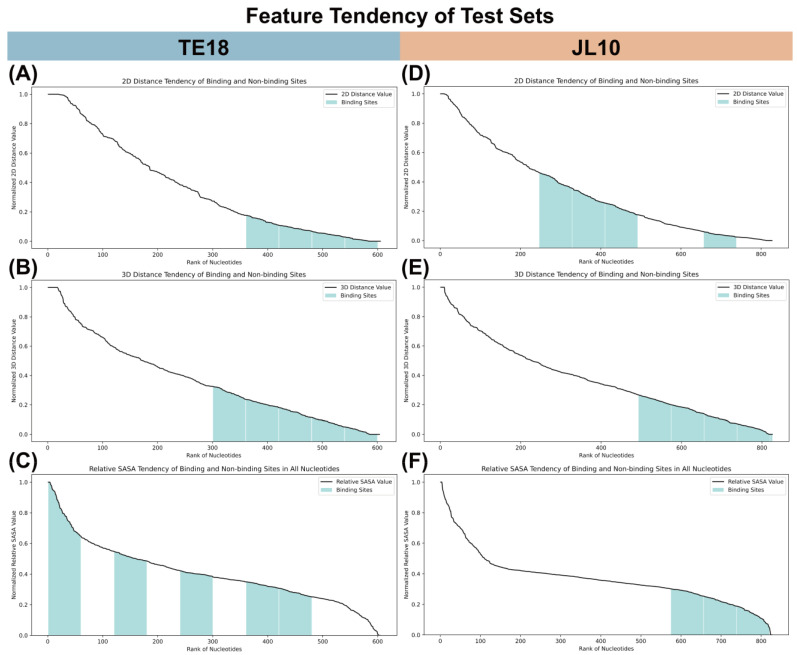
Feature tendency of binding and non-binding sites in TE18 and JL10 test sets. Binding site groups are labeled in green. (**A**) 2D distance tendency of nucleotides in TE18. (**B**) 3D distance tendency of nucleotides in TE18. (**C**) Relative SASA tendency of nucleotides in TE18. (**D**) 2D distance tendency of nucleotides in JL10. (**E**) 3D distance tendency of nucleotides in JL10. (**F**) Relative SASA tendency of nucleotides in JL10.

First, we calculated the sum of Euclidean distances of the tertiary structure from the C1’ atom of each nucleotide to all other nucleotides’ C1’ atoms, similar to Rsite, with the resulting trends shown in Figure 3A,D. In TE18, four of the ten groups were identified as binding-site groups, ranking first to fourth in ascending order of these sums, indicating that binding sites tend to have smaller tertiary-structure distances. In JL10, four groups also appeared as binding-site groups, ranking second, fifth, sixth, and seventh, indicating a modest tendency toward shorter distances. For secondary structure distances, we extracted 2D coordinates from the RNAfold output files, as Rsite2 did. Figure 3B,E show that in TE18, five groups were recognized as binding sites, ranking first to fifth, while in JL10, four groups ranked first to fourth, indicating a tendency for binding sites in both to have smaller secondary-structure distance sums. Relative SASA was calculated using freesasa [75], with the tendencies shown in Figure 3C,F. In JL10, three binding-site groups ranked first to third, showing a clear tendency toward lower values, whereas no clear trend was observed in TE18. This suggests that relying solely on SASA is insufficient to distinguish binding from non-binding sites; combining multiple features is essential for accurately characterizing nucleotide properties.

**Figure 4:**
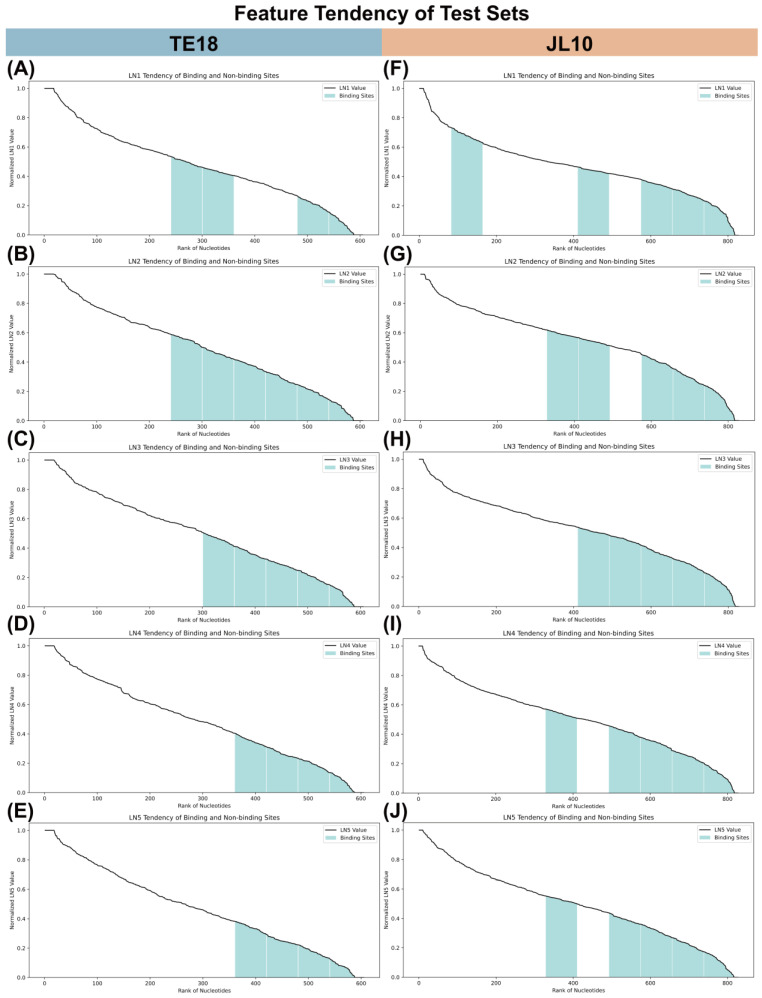
Feature tendency of binding and non-binding sites in TE18 and JL10 test sets. Binding site groups are labeled in green. Tendencies of nucleotides in TE18 across (**A**) LN1, (**B**) LN2, (**C**) LN3, (**D**) LN4, (**E**) LN5. Tendencies of nucleotides in JL10 across (**F**) LN1, (**G**) LN2, (**H**) LN3, (**I**) LN4, (**J**) LN5.

As mentioned earlier, LN serves as a feature that describes how concave or convex nucleotides are. An LN value near 1 suggests the nucleotide has a more convex surface, while a value near 0 indicates a more concave surface. We computed LN using five different σ values, similarly to ZeSTa, to account for nucleotides at various distances from the target, offering a thorough overview of surface concavity and convexity. Figure 4A,F display the LN1 results computed with σ = 0, which mainly reflect the influence of spatially neighboring nucleotides. In TE18, the four binding-site groups are ranked in increasing order of their values, from first to fourth, while in JL10, the five binding-site groups are ranked first, second, third, fifth, and ninth. The remaining panels of Figure 4 show the results of LN2, LN3, LN4, and LN5 as σ was gradually increased, thereby giving more weight to distant nucleotides. Binding-site groups tend to cluster within the first five in ascending order, indicating that whether the computation emphasizes nearby or distant nucleotides, binding sites generally have smaller LN values. Additionally, in both TE18 and JL10, binding sites often display lower LN values, suggesting they tend to have more concave surfaces. Both LN and SASA are features used to describe the surface properties of nucleotides. In JL10, binding sites tend toward smaller SASA values, and in both JL10 and TE18, they consistently have lower LN values, reinforcing the tendency toward concave surfaces. This is because nucleotides with convex surfaces are more exposed to solvent, resulting in larger SASA, while those with concave surfaces are less accessible to solvent probes, leading to lower SASA values.

We also analyzed the tendencies of network and pocket features. Figures S1–S5 illustrates the five network features of nucleotides in TE18 and JL10. It shows that binding sites in both samples display similar tendencies. Figure S1A,B present the local feature degree, where the three binding-site groups in TE18 ranked first, second, and fifth, while the four groups in JL10 ranked within the top four in descending order. Results for neighborhood connectivity, closeness, and betweenness are shown in Figures S2–S4, indicating that binding sites tend to have higher values for these three features. Conversely, Figure S5 reveals that the five binding-site groups in TE18 ranked first, second, third, fifth, and seventh in ascending order, and those in JL10 ranked within the top five. Binding sites generally show higher degree and neighborhood connectivity, suggesting they are often situated in the core topological regions of nucleotide networks with strong local connectivity and dense links. For the global network features, binding sites tend to have higher closeness and betweenness scores and lower eccentricity, implying they are closer to the network’s geometric center and participate more in shortest paths, thus playing a vital role in information flow. Additionally, their distances to the most distant nodes are shorter, indicating they are positioned nearer the network center rather than at the periphery.

Figures S6–S10 depicts the trends in pocketness, a measure of the size and depth of nucleotide-formed pockets. We employed the same approach as ZeSTa for calculating pocketness, applying the Ghecom dual-probe method with a large default probe radius. We then measured the pocketness of each nucleotide at smaller radii of 1.5, 2.0, 2.5, 3.0, and 3.5 Å, labeled as PKT1 through PKT5. This multi-radius method captures pocket formation at different scales. Figures S6A–S10A show results for TE18, while Figures S6B–S10B display those for JL10. A pocketness close to 1 indicates larger, deeper pockets. In both test sets, binding-site nucleotides tend to cluster within the top five values, especially in PKT1–3, suggesting they form larger, deeper pockets suitable for small molecule binding. At these scales, binding sites are more likely to form sizable pockets compared to non-binding regions. Conversely, at PKT4 and PKT5, many nucleotides have pocketness values of 0, meaning the probes were too large for pocket detection at these scales. In TE18, this is evident from Figures S9A and S10A, and in JL10 from Figures S9B and S10B. The binding site tendency is less clear in PKT4 and PKT5, so focusing on PKT1-3 may be more effective.

#### 4.2.3. Performance Analysis

Four metrics are used to evaluate the performances of methods, namely, precision, recall, Matthews correlation coefficient (MCC) and the area under the receiver operating characteristic curve (AUC). Precision, recall, MCC can be calculated as follows:

(8) $$Precision=\frac{\left|TP\right|}{\left(\left|TP\right|+\left|FP\right|\right)}$$

(9) $$Recall=\frac{\left|TP\right|}{\left(\left|TP\right|+\left|FN\right|\right)}$$

(10) $$MCC=\frac{TP\times TN-FP\times FN}{\sqrt{\left(TP+FP\right)\left(TP+FN\right)\left(TN+FP\right)\left(TN+FN\right)}}$$

where TP is true positive and represents the number of true binding nucleotides within the predicted binding nucleotides. FP is false positive and represents the number of false binding nucleotides within the predicted binding nucleotides. TN is the true negative and represents the number of real non-binding nucleotides with the predicted non-binding nucleotides. FN is a false negative representing the number of binding nucleotides with the predicted non-binding nucleotides.

The precision performance of methods on TE18 is shown in Figure 2B. Results for Rsite, Rsite2, RBind, and RNAsite were obtained from the RNAsite publication, whereas results for the other methods were derived from their respective original papers. Among physics-based methods, RBind (0.655), which incorporates two network features, outperforms the distance-based Rsite (0.449) and Rsite2 (0.370). As for the AI-based methods, the precision of MultiModRLBP (0.644) is approximately 1.7% lower than that of RBind (0.655), while RNAsite (0.675), RLBind (0.681), RNetsite (0.701), ZeSTa (0.729), and RLsite (0.712) all outperform the physics-based approaches. Figure 2C presents the precision performance of methods on JL10. The results for Rsite, Rsite2, RBind were from the ZeSTa publication, the RNAsite results were obtained from its web server in August 2025, and the RNetsite results were generated in August 2025 using the V2 package provided on its website. The remaining results were from their respective publications. The precision of all methods decreased in RNAs with more complex structures, but the overall trend remains consistent. RBind (0.433) still outperforms Rsite (0.295) and Rsite2 (0.338). Meanwhile, the AI-based methods RNAsite (0.668), RNetsite (0.458), ZeSTa (0.549), and RLsite (0.622) show precision improvements over RBind of 54.3%, 5.8%, 26.8%, and 43.6%, respectively. The other metrics for the various methods on the two test sets are presented in Table 3 and Table 4.

In the tendency figures shown in Figure 3A,B,D,E, binding sites consistently show smaller values in both tertiary-structure-based and secondary-structure-based distance sums. This highlights the potential of 2D/3D distance sums to characterize binding site properties. Consequently, distance-sum-based methods, such as Rsite and Rsite2, effectively predict small molecule binding sites in RNA. However, calculating distance sums by giving equal weight to all nucleotides can introduce significant noise from irrelevant interactions. Additionally, relying only on distance sums cannot capture multi-body interactions between nucleotides or distinguish between long- and short-range interactions. RBind utilizes network modeling to integrate local and global features, enabling it to represent multi-body interactions and differentiate between different interaction types. Network topological features help identify key nodes and reduce noise in the interaction network. As a result, RBind outperforms Rsite and Rsite2. Still, these three physics-based methods are limited to a few features and cannot incorporate more diverse and complementary information, which restricts their predictive capabilities.

Machine learning and deep learning algorithms can effectively integrate multiple features. On both test sets, AI-based methods outperform physics-based methods by leveraging various features. Among methods relying solely on network features, RNetsite uses machine learning algorithms to incorporate more complementary network features. On the TE18/JL10 test sets, RNetsite (0.675/0.458) achieved higher precision than RBind (0.655/0.433). Although RNetsite considers complementary network features, its performance is lower than methods with a more diverse feature set, such as ZeSTa (0.729/0.549), due to its lack of other feature perspectives like sequence information. Especially on JL10, which has RNA with more complex structures, RNetsite’s precision is only 5.8% higher than RBind’s, compared to ZeSTa’s 26.8% improvement over RBind. ZeSTa achieved the highest precision on TE18 but underperformed on JL10 compared to RNAsite (0.675/0.668) and RLsite (0.712/0.622), likely due to the high structural sensitivity of its geometric features. Like ZeSTa, RLsite combines sequence-based, geometric-based, and network-based features, and additionally introduces energy features to analyze nucleotides through interaction energy, which ZeSTa does not include. These findings suggest that effective RNA–small molecule binding site prediction requires not only complementary features but also a diverse combination of features.

Since features derived from RNA sequences are independent of RNA structures, incorporating sequence-based features offers a more comprehensive characterization of nucleotides. Therefore, most AI methods rely on sequence-based features. RNA sequence information can be extracted via two approaches: using MSA-DCA (as seen in RNAsite, RLBind, ZeSTa, and RLsite) and utilizing RNA LLMs (as in MultiModRLBP and RNABind). MSA-DCA examines relationships between nucleotides from an evolutionary standpoint. However, a limitation of MSA-DCA is that many RNA sequences lack sufficient homologous sequences, which restricts the information it can provide. Conversely, RNA LLMs are trained on large-scale sequence datasets and do not require searching for homologs during application, making them more efficient. Notably, RNABind trained separate models using embeddings from eight different LLMs and observed that their predictive performances varied. Therefore, choosing the right RNA LLM to represent sequence information is crucial for accurate predictions.

Besides sequence-based features, methods for predicting RNA–small molecule binding sites utilize various structure-based features, which are classified into four groups: network, geometry, energy, and secondary structure. Combining features from the same group can enhance the description of nucleotide properties, while integrating features from different groups provides a more complete characterization. For instance, network features derived from tertiary structure models describe binding sites via network topology. These sites are often located in the dense core of the local nucleotide network and are closer to the network center, thereby facilitating their role in communication. While network features alone offer valuable insights, they have limitations when it comes to describing the intrinsic properties of individual nucleotides. To address this, many methods incorporate geometric features, such as SASA, which indicates solvent exposure, showing a lower tendency, mainly on JL10, with no clear pattern on TE18. Methods such as RNAsite, ZeSTa, and RLsite combine SASA with LN or pocketness to better describe the geometric traits of nucleotides. This combined approach helps identify binding sites in pocket regions with more concave surfaces, leading to lower solvent exposure. Some methods further improve prediction accuracy by adding energy features (RLsite) and secondary structure features (ZeSTa), demonstrating the benefit of combining features across different categories. Ultimately, effective nucleotide structure characterization depends on integrating diverse features within and across categories for a thorough understanding.

## 5. Future Directions

Existing approaches for predicting RNA–small molecule binding sites employ various features and models. Overall, AI-based methods, which integrate diverse features to characterize nucleotides, tend to outperform physics-based techniques that rely on isolated strategies. The effectiveness of AI models largely depends on the quality and quantity of the training data used. Therefore, increasing the amount of RNA data that does not overlap with test sets for training can enhance predictive accuracy. By analyzing the structural and sequence similarities within the current training datasets, we observed many RNAs that are structurally alike, and a few RNA chains that share numerous homologous sequences across different datasets [76,77]. To expand the amount of structural data available for training AI models with improved accuracy, tools like AlphaFold can be utilized to perform enhanced sampling, such as introducing mutations and creating conformational ensembles [78,79,80].

Additionally, selecting the right combination of features is essential for effectively characterizing nucleotides. However, adding more feature types increases computational costs. It is essential to identify a feature set that is both diverse and complementary, yet computationally manageable. Previous studies highlight that including geometric features like LN, pocket, and interaction-based energy features is essential for improving the model’s predictive accuracy, as these features display clear distribution differences between binding and non-binding sites [22,25,28]. Nonetheless, while structure-based refined features effectively capture RNA’s structural properties, their high sensitivity to structural variations often causes a notable drop in predictive performance when input structures deviate from the native conformation. Conversely, sequence-based features, which do not depend on detailed structural information, or coarse-grained network features less sensitive to structural changes respond more consistently to structural perturbations and tend to be more robust under variations [24].

Furthermore, choosing an appropriate AI algorithm can maximize the use of limited data to identify underlying patterns. For simple or short structures, machine learning models with fewer parameters or lightweight deep learning models, combined with a single feature type, can deliver satisfactory predictive results. However, for more complex structures, advanced model architectures are necessary, involving multiple modules and multimodal features to fully represent RNA from sequence to structure, thus improving prediction accuracy. Addressing these factors can lead to more accurate predictions of RNA–small molecule binding sites.

The flexibility of RNA structure is a critical aspect in the development of RNA–small molecule drugs [69]. This flexibility manifests not only in the molecule’s dynamic conformational changes but also in the structural shifts that occur before and after ligand binding, reflecting differences between the unbound and bound states. The secondary and tertiary structures can vary significantly between these states, influencing small-molecule binding. Therefore, considering RNA’s structural flexibility is essential in practical drug development. However, most current binding-site prediction methods mainly depend on RNA conformations derived from known RNA–small molecule complexes (the bound state), often overlooking the unbound state. This restriction makes applying predicted sites to guide drug development more challenging. Future research should emphasize RNA’s structural flexibility to better mirror real-world scenarios. For example, by incorporating conformational ensembles of RNA structures obtained from molecular dynamics simulations or NMR data, it is possible to include information on the dynamic structural changes of RNA and account for its structural flexibility [81,82]. As the accuracy of RNA–small molecule binding site prediction improves, it will lay a robust foundation and create new opportunities for designing drugs that target RNA.

## Figures and Tables

**Table 1:** Methods for predicting RNA-small molecule binding sites. Input refers to the input information of the model, where seq represents sequence and 3D structure refers to tertiary structure. Feature combination refers to the feature types the method utilizes, MSA stands for multiple sequence alignment, SS stands for secondary structure. Model refers to the computing algorithm, where RF refers to Random Forest, CNN refers to Convolutional Neural Network, XGB refers to Extreme Gradient Boosting, LGBM refers to Light Gradient Boosting Machine, ResNet refers to Residual Convolutional Network, RGCN refers to Relational Graph Convolutional Network, EGNN refers to E(3)-Equivariant Graph Neural Network, 3DCNN refers to 3D Convolutional Neural Network, BiLSTM refers to Bidirectional Long Short-term Memory Network. Available records the available source or web server of method. Ref records the paper related to the method.

Name	Input	Feature Combination	Model	Available	Ref.
Rsite	3D structure	3D distance	Distance	http://www.cuilab.cn/rsite (accessed on 20 August 2025)	[19]
Rsite2	seq	2D distance	Distance	https://www.cuilab.cn/rsite2 (accessed on 20 August 2025)	[20]
RBind	3D structure	3D distance	Distance	http://zhaoserver.com.cn/RBinds/RBinds.html (accessed on 20 August 2025)	[21]
RNAsite	seq, 3D structure	MSA, Geometry, Network	RF	https://yanglab.qd.sdu.edu.cn/RNAsite/ (accessed on 20 August 2025)	[22]
RLBind	seq, 3D structure	MSA, Geometry, Network	CNN	https://github.com/KailiWang1/RLBind (accessed on 20 August 2025)	[23]
RNetsite	3D structure	Network	RF, XGB, LGBM	http://zhaoserver.com.cn/RNet/RNet.html (accessed on 20 August 2025)	[24]
ZHmolReSTasite	seq, 3D structure	MSA, SS, Geometry, Network	ResNet	http://zhaoserver.com.cn/ZHmol/ZHmolReSTasite/ZHmolReSTasite.html (accessed on 20 August 2025)	[25]
MultiModRLBP	seq, 3D structure	LLM, Geometry, Network	CNN, RGCN	https://github.com/lennylv/MultiModRLBP (accessed on 20 August 2025)	[26]
RNABind	seq, 3D structure	LLM	EGNN	https://github.com/jaminzzz/RNABind (accessed on 20 August 2025)	[27]
RLsite	seq, 3D structure	MSA, Geometry, Energy	3DCNN, BiLSTM	https://github.com/fine1231/RLsite (accessed on 20 August 2025)	[28]

**Table 2:** Features utilized in methods for predicting RNA–small molecule binding sites. Name refers to the feature names, where MSA represents multiple sequence alignment, plmDCA represents pseudolikelihood maximization direct coupling analysis, LLM embedding represents large language model embedding, SASA represents solvent accessible surface area. Type refers to the categories of RNA information required for feature computation. Brief principle refers to concise explanations of the feature characteristics. Available refers to the available code or website in which the feature is obtained in the related methods. Related method refers to the method that utilizes the feature.

Name	Type	Brief Principle	Available	Related Method
MSA	seq	MSA reveals sequence similarity and evolutionary relationship	BLASTN: https://blast.ncbi.nlm.nih.gov/ (accessed on 20 August 2025)	RNAsite, RLBind, ZeSTa, RLsite
plmDCA	seq	plmDCA focuses on direct interactions within nucleotide mutual information	ZeSTa code: http://zhaoserver.com.cn/ZHmol/ZHmolReSTasite/ZHmolReSTasite.html (accessed on 20 August 2025)	RLBind, ZeSTa, RLsite
			RLsite code: https://github.com/fine1231/RLsite (accessed on 20 August 2025)	
			Consurf-DB webserver: http://consurf.tau.ac.il (accessed on 20 August 2025)	
LLM embedding	seq	LLM embeddings capture the distribution patterns of RNA sequences and their contextual semantic information.	LucaOne: https://github.com/LucaOne/LucaOneApp (accessed on 20 August 2025)	MultiModRLBP, RNABind
			RNA-FM: https://github.com/ml4bio/RNA-FM (accessed on 20 August 2025)	
			ProtRNA: https://github.com/roxie-zhang/ProtRNA (accessed on 20 August 2025)	
			RiNALMo: https://github.com/lbcb-sci/RiNALMo (accessed on 20 August 2025)	
			ERNIE-RNA: https://github.com/Bruce-ywj/ERNIE-RNA (accessed on 20 August 2025)	
			RNAErnie: https://github.com/CatIIIIIIII/RNAErnie (accessed on 20 August 2025)	
			RNA-MSM: https://github.com/yikunpku/RNA-MSM (accessed on 20 August 2025)	
LLM embedding	seq	LLM embeddings capture the distribution patterns of RNA sequences and their contextual semantic information.	RNABERT: https://github.com/mana438/RNABERT (accessed on 20 August 2025)	MultiModRLBP, RNABind
Secondary Structure Region	secondary structure	Regional classification can distinguish the loop types of the secondary structure and the stem regions where nucleotides are located	RNAstat: https://github.com/RNA-folding-lab/RNAStat (accessed on 20 August 2025)	ZeSTa
SASA	3D structure	SASA describes the measure of surface exposure of nucleotides.	RNAsol: https://yanglab.qd.sdu.edu.cn/RNAsol/ (accessed on 20 August 2025)	RNAsite, RLBind, ZeSTa, MultiModRLBP, RLsite
			Freesasa, POPS	
Degree	3D structure	Degree reflects the direct interactions between a nucleotide node and its neighbors.	RLBind code: https://github.com/KailiWang1/ (accessed on 20 August 2025)	RBind, RNAsite, RNetsite, RLBind, ZeSTa, MultiModRLBP,RLsite
			RNetsite code: http://zhaoserver.com.cn/RNet/RNet.html (accessed on 20 August 2025)	
			ZeSTa code: http://zhaoserver.com.cn/ZHmol/ZHmolReSTasite/ZHmolReSTasite.html (accessed on 20 August 2025)	
			RLsite code: https://github.com/fine1231/RLsite (accessed on 20 August 2025)	
Neighborhood Connectivity	3D structure	Neighborhood connec-tivity characterizes the average number of connections of the neighboring nodes of a nucleotide node.	RNetsite code: http://zhaoserver.com.cn/RNet/RNet.html (accessed on 20 August 2025)	RNetsite
Closeness	3D structure	Closeness measures the average distance from a nucleotide node to all other nodes.	RLBind code: https://github.com/KailiWang1/ (accessed on 20 August 2025)	RBind, RNAsite, RNetsite, RLBind, ZeSTa, MultiModRLBP,RLsite
			RNetsite code: http://zhaoserver.com.cn/RNet/RNet.html (accessed on 20 August 2025)	
			ZeSTa code: http://zhaoserver.com.cn/ZHmol/ZHmolReSTasite/ZHmolReSTasite.html (accessed on 20 August 2025)	
Closeness	3D structure	Closeness measures the average distance from a nucleotide node to all other nodes.	RLsite code: https://github.com/fine1231/RLsite (accessed on 20 August 2025)	RBind, RNAsite, RNetsite, RLBind, ZeSTa, MultiModRLBP,RLsite
Eccentricity	3D structure	Eccentricity measures the maximum distance from a nucleotide node to all other nodes in the network.	RNetsite code: http://zhaoserver.com.cn/RNet/RNet.html (accessed on 20 August 2025)	RNetsite
Betweenness	3D structure	Betweenness centrality measures how frequently a node appears on all shortest paths in the network.	RNetsite code: http://zhaoserver.com.cn/RNet/RNet.html (accessed on 20 August 2025)	RNetsite
Laplacian Norm	3D structure	The Laplacian Norm describes the concavity and convexity of nucleotides.	ZeSTa code: http://zhaoserver.com.cn/ZHmol/ZHmolReSTasite/ZHmolReSTasite.html (accessed on 20 August 2025)	RNAsite, ZeSTa RLsite
			RLsite code: https://github.com/fine1231/RLsite (accessed on 20 August 2025)	
Pocket	3D structure	A pocket is an internal cavity within the tertiary structure of RNA.	Ghecom: https://pdbj.org/ghecom/README_ghecom.html (accessed on 20 August 2025)	ZeSTa
Interaction Energy	3D structure	The interaction between RNA and small molecules maintains the stability of the complex structure.	RLsite code: https://github.com/fine1231/RLsite (accessed on 20 August 2025)	RLsite

**Table 3:** Model performance on easy-task test set TE18. The evaluation metrics include precision, recall, MCC and AUC, where MCC refers to Matthews Correlation Coefficient and AUC refers to Area Under the Receiver Operating Characteristic Curve. The results of Rsite, Rsite2, RBind, and RNAsite are derived from the report of RNAsite paper, while the results of the other methods are derived from their respective papers. The best performance is bolded, and the second-best is underlined.

Name	Precision	Recall	MCC	AUC
Rsite	0.449	0.288	0.071	0.509
Rsite2	0.370	0.214	0.010	0.474
RBind	0.655	0.173	0.187	0.559
RNAsite	0.675	0.263	0.253	0.776
RLBind	0.681	0.345	0.324	0.720
RNetsite	0.701	0.357	0.307	-
ZHmolReSTasite	**0.729**	0.379	0.327	0.709
MultiModRLBP	0.644	**0.523**	**0.378**	**0.780**
RNABind	-	-	-	0.737
RLsite	0.712	0.392	0.335	0.740

**Table 4:** Model performance on challenging-task test set JL10. The evaluation metrics include precision, recall, MCC and AUC, where MCC refers to Matthews Correlation Coefficient and AUC refers to Area Under the Receiver Operating Characteristic Curve. The results of Rsite, Rsite2, RBind, and ZHmolReSTasite are reported in the ZHmolReSTasite paper, the results of RNAsite are obtained from the RNAsite web server (August 2025), the results of RNetsite are obtained from the RNetsite V2 package on its website (August 2025), and the results of the other methods are derived from their respective papers. The best performance is bolded, and the second-best is underlined.

Name	Precision	Recall	MCC	AUC
Rsite	0.295	0.194	−0.046	0.477
Rsite2	0.338	0.131	0.007	0.504
RBind	0.433	0.142	0.083	0.532
RNAsite	**0.668**	**0.327**	**0.323**	0.637
RNetsite	0.458	0.136	0.115	0.540
ZHmolReSTasite	0.549	0.296	0.211	0.592
RNABind	-	-	-	0.667
RLsite	0.622	0.320	0.286	**0.700**

## Data Availability

No new data were created or analyzed in this study. Data sharing is not applicable.

## Supplementary Materials

The following supporting information can be downloaded at: https://www.mdpi.com/article/10.3390/ph18101593/s1, Figure S1: Degree tendencies of binding and non-binding sites in (A) TE18 and (B) JL10 test sets. Binding site groups are labeled in green; Figure S2: Neighborhood connectivity tendencies of binding and non-binding sites in (A) TE18 and (B) JL10 test sets. Binding site groups are labeled in green; Figure S3: Closeness tendencies of binding and non-binding sites in (A) TE18 and (B) JL10 test sets. Binding site groups are labeled in green; Figure S4: Betweenness tendencies of binding and non-binding sites in (A) TE18 and (B) JL10 test sets. Binding site groups are labeled in green; Figure S5: Eccentricity tendencies of binding and non-binding sites in (A) TE18 and (B) JL10 test sets. Binding site groups are labeled in green; Figure S6: PKT1 tendencies of binding and non-binding sites in (A) TE18 and (B) JL10 test sets. Binding site groups are labeled in green; Figure S7: PKT2 tendencies of binding and non-binding sites in (A) TE18 and (B) JL10 test sets. Binding site groups are labeled in green; Figure S8: PKT3 tendencies of binding and non-binding sites in (A) TE18 and (B) JL10 test sets. Binding site groups are labeled in green; Figure S9: PKT4 tendencies of binding and non-binding sites in (A) TE18 and (B) JL10 test sets. Binding site groups are labeled in green; Figure S10: PKT5 tendencies of binding and non-binding sites in (A) TE18 and (B) JL10 test sets. Binding site groups are labeled in green; Figure S11: Feature distribution in 2D distance, 3D distance, and RSASA of binding and non-binding sites in (A) TE18 and (B) JL10 test sets. Binding sites are labeled in purple, and non-binding sites are labeled in green; Figure S12: Feature distribution in Laplacian Norm (LN) of binding and non-binding sites in (A) TE18 and (B) JL10 test sets. Binding sites are labeled in purple, and non-binding sites are labeled in green; Figure S13: Feature distribution in network features (degree, neighborhood connectivity, closeness, betweenness, and eccentricity) of binding and non-binding sites in (A) TE18 and (B) JL10 test sets. Binding sites are labeled in purple, and non-binding sites are labeled in green; Figure S14: Feature distribution in pocketness of binding and non-binding sites in (A) TE18 and (B) JL10 test sets. Binding sites are labeled in purple, and non-binding sites are labeled in green; Figure S15: Sequence and structure similarity of three training sets (TR60, trainRLBP, and training set of RNABind). RLBP represents trainRLBP, and RNAB stands for the training set of RNABind. Each dot depicts an RNA in the dataset; the x-coordinate shows the maximum TM-score between the query RNA and any structure in the other dataset, while the y-coordinate indicates the number of RNA chains in the other dataset that fall into the same cluster as the query RNA.

## Abbreviations

LLM: Large language model; MSA, Multiple sequence alignment; DCA, Direct coupling analysis; ResNet, Residual network; PKT, Pocket; SASA, Solvent accessible surface area; LN, Laplacian norm; AI, Artificial intelligence; EGNN, E(3)-equivariant graph neural network.
