# Supplementary material for: Advances in Methods for Accurate Prediction of RNA–Small Molecule Binding Sites: From Isolated to AI-Integrated Strategies
Source: Pharmaceuticals (Basel). 2025 Oct 21;18(10):1593. doi: 10.3390/ph18101593 (PMC12567027; doi:10.3390/ph18101593)
Supplement: Supplementary file 1 [file pharmaceuticals-18-01593-s001.zip › pharmaceuticals-3924978-supplementary.pdf]

Advances in Methods for Accurate Prediction of RNA-Small Molecule Binding Sites: From Isolated to AI-integrated Strategies

*Jiaming Gao, Chen Zhuo, Chengwei Zeng, Haoquan Liu, and Yunjie Zhao**

Institute of Biophysics and Department of Physics, Central China Normal University, Wuhan, 430079, China

Figures

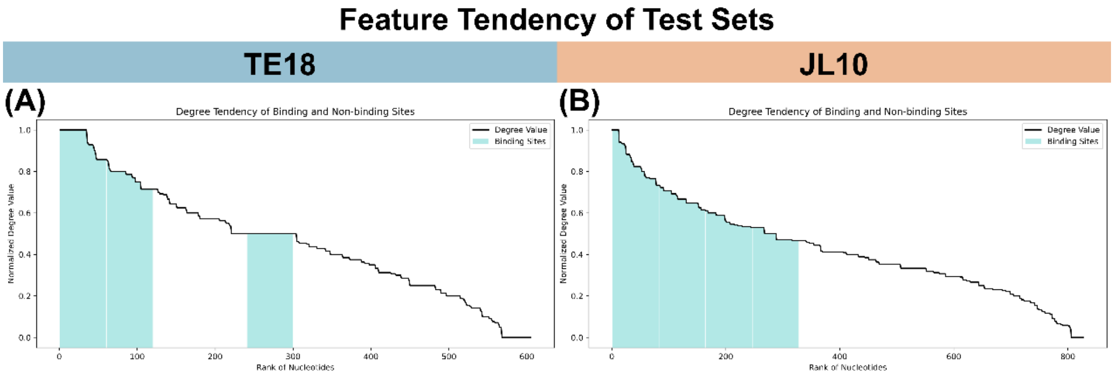

Figure S1. Degree tendencies of binding and non-binding sites in (A) TE18 and (B) JL10 test sets. Binding site groups are labeled in green.

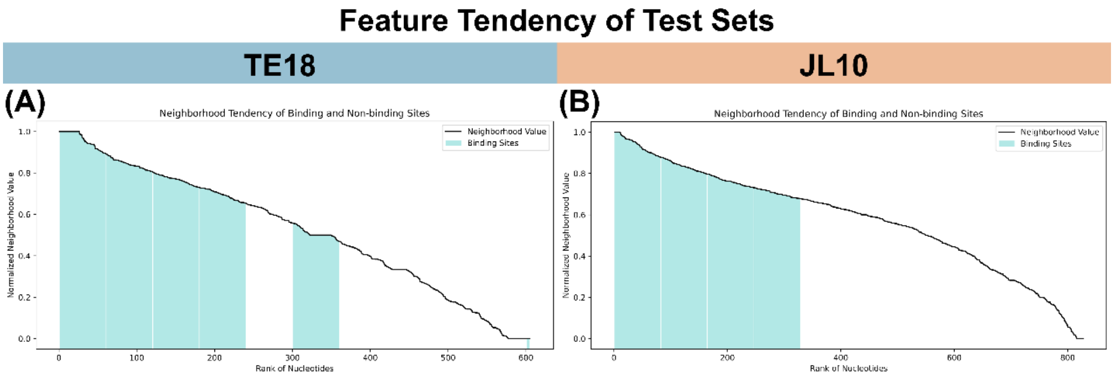

Figure S2. Neighborhood connectivity tendencies of binding and non-binding sites in (A) TE18 and (B) JL10 test sets. Binding site groups are labeled in green.

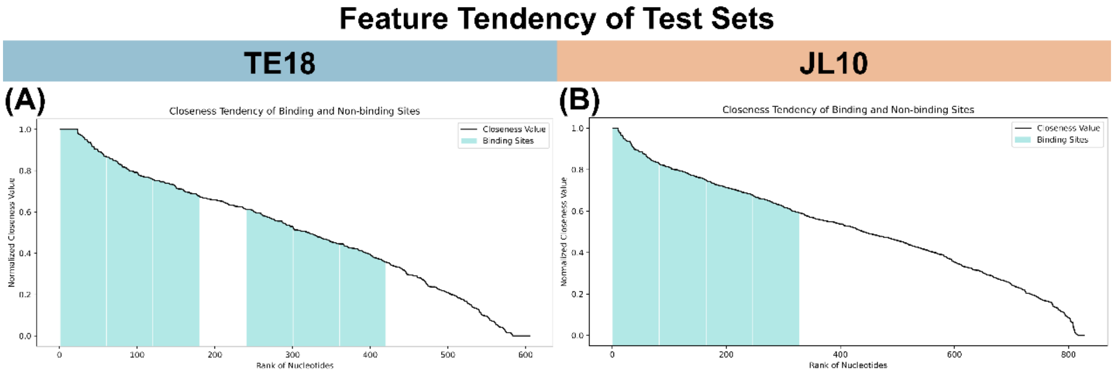

Figure S3. Closeness tendencies of binding and non-binding sites in (A) TE18 and (B) JL10 test sets. Binding site groups are labeled in green.

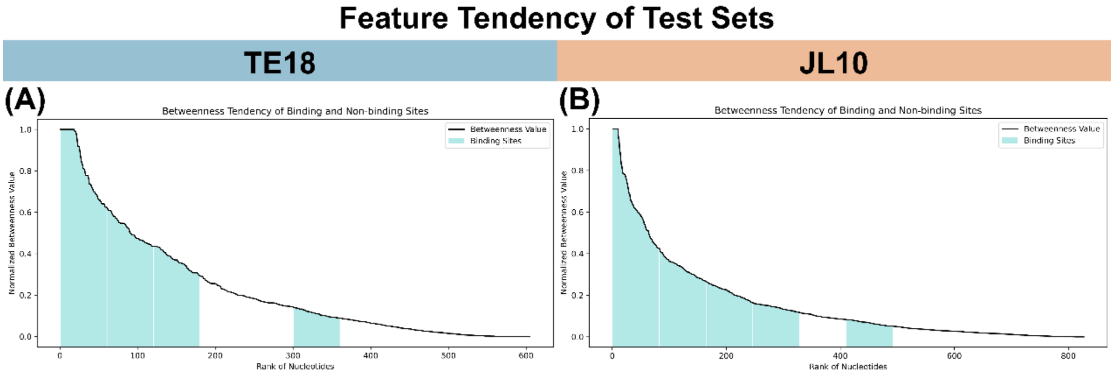

Figure S4. Betweenness tendencies of binding and non-binding sites in (A) TE18 and (B) JL10 test sets. Binding site groups are labeled in green.

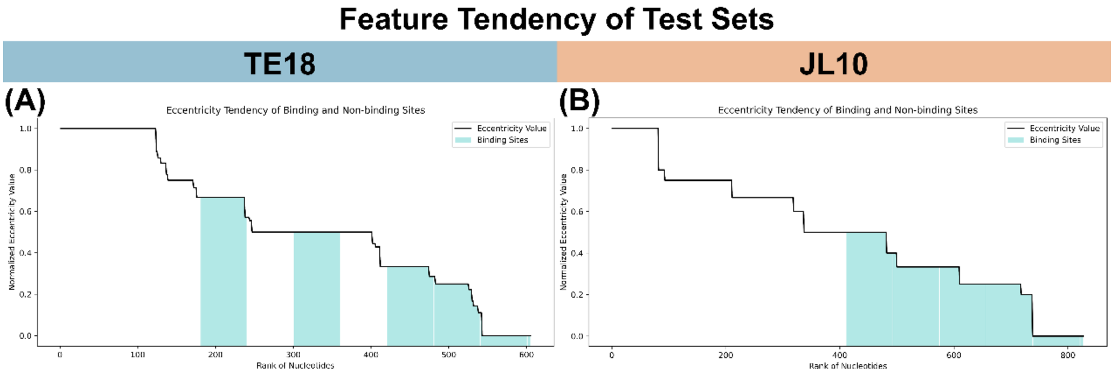

Figure S5. Eccentricity tendencies of binding and non-binding sites in (A) TE18 and (B) JL10 test sets. Binding site groups are labeled in green.

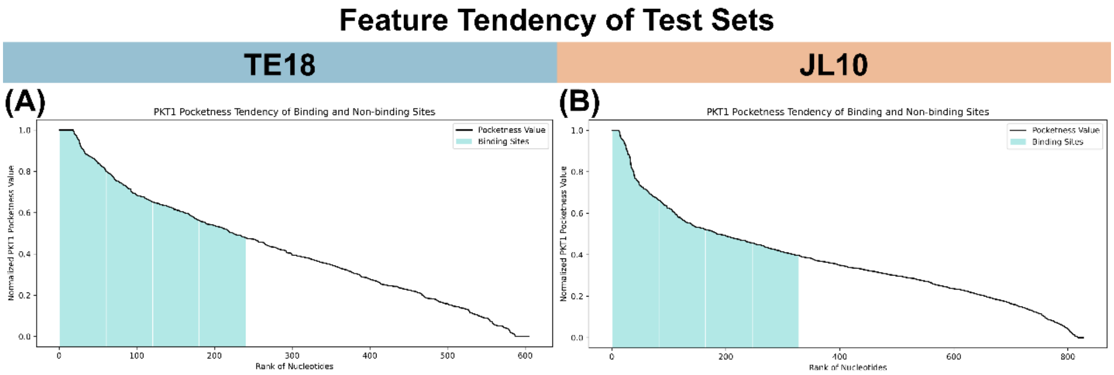

Figure S6. PKT1 tendencies of binding and non-binding sites in (A) TE18 and (B) JL10 test sets. Binding site groups are labeled in green.

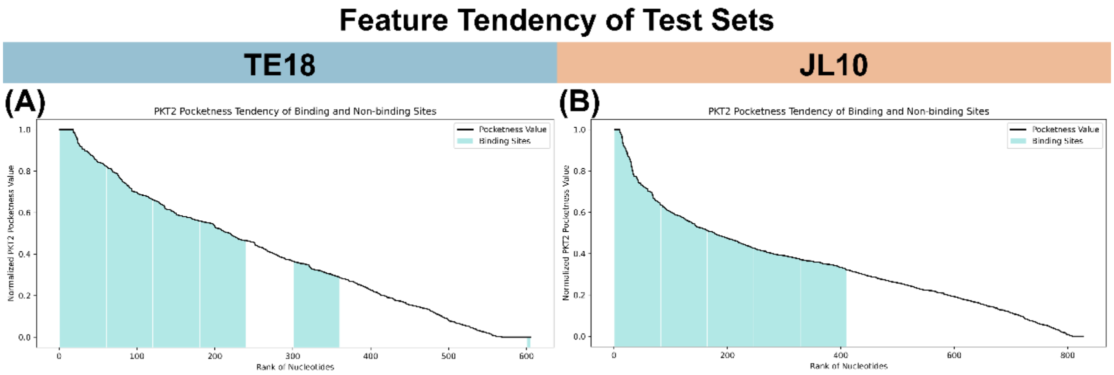

Figure S7. PKT2 tendencies of binding and non-binding sites in (A) TE18 and (B) JL10 test sets. Binding site groups are labeled in green.

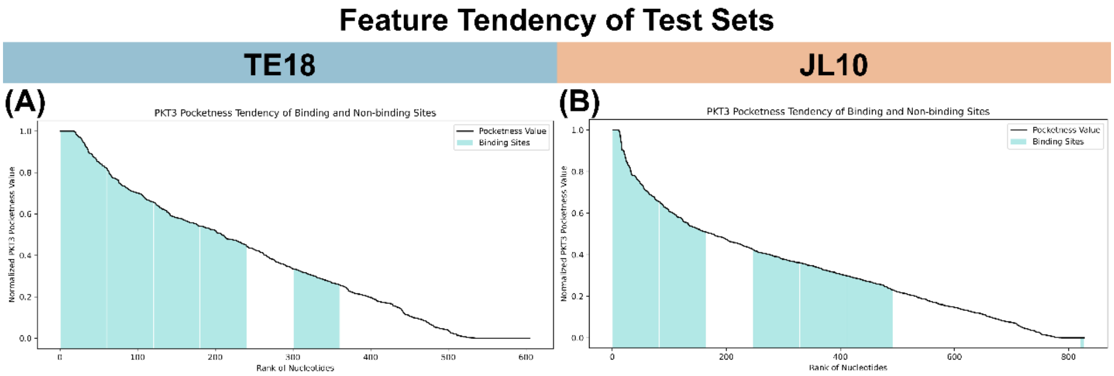

Figure S8. PKT3 tendencies of binding and non-binding sites in (A) TE18 and (B) JL10 test sets. Binding site groups are labeled in green.

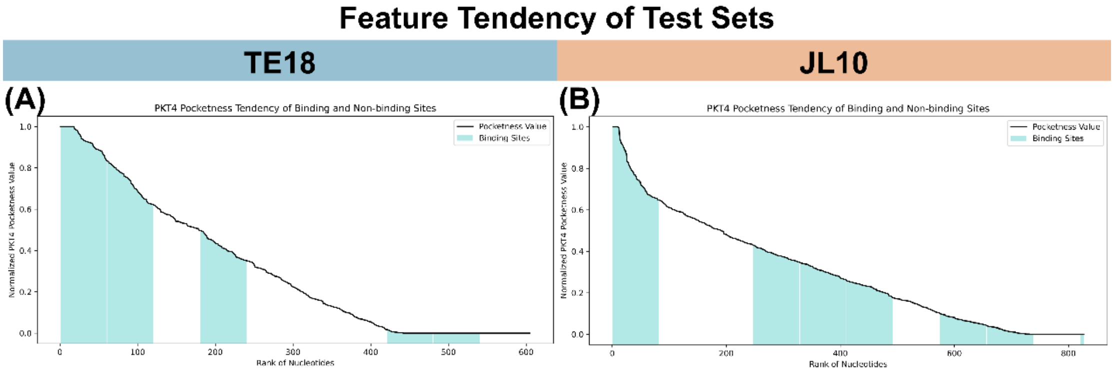

Figure S9. PKT4 tendencies of binding and non-binding sites in (A) TE18 and (B) JL10 test sets. Binding site groups are labeled in green.

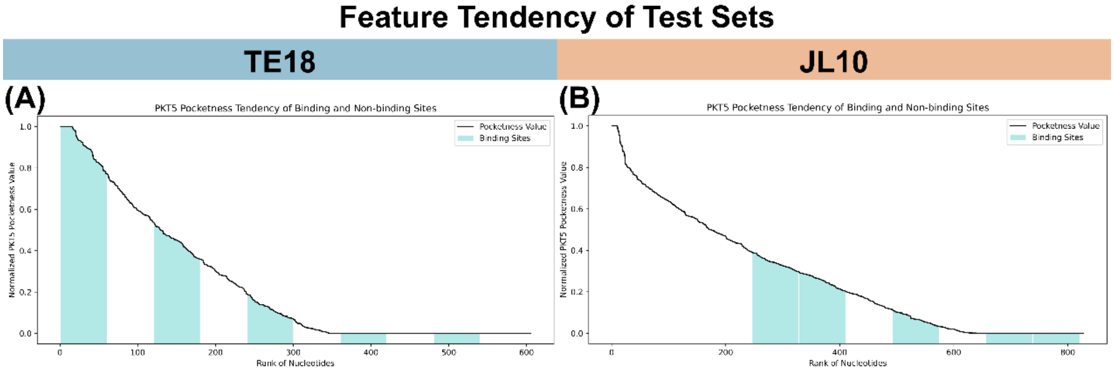

Figure S10. PKT5 tendencies of binding and non-binding sites in (A) TE18 and (B) JL10 test sets. Binding site groups are labeled in green.

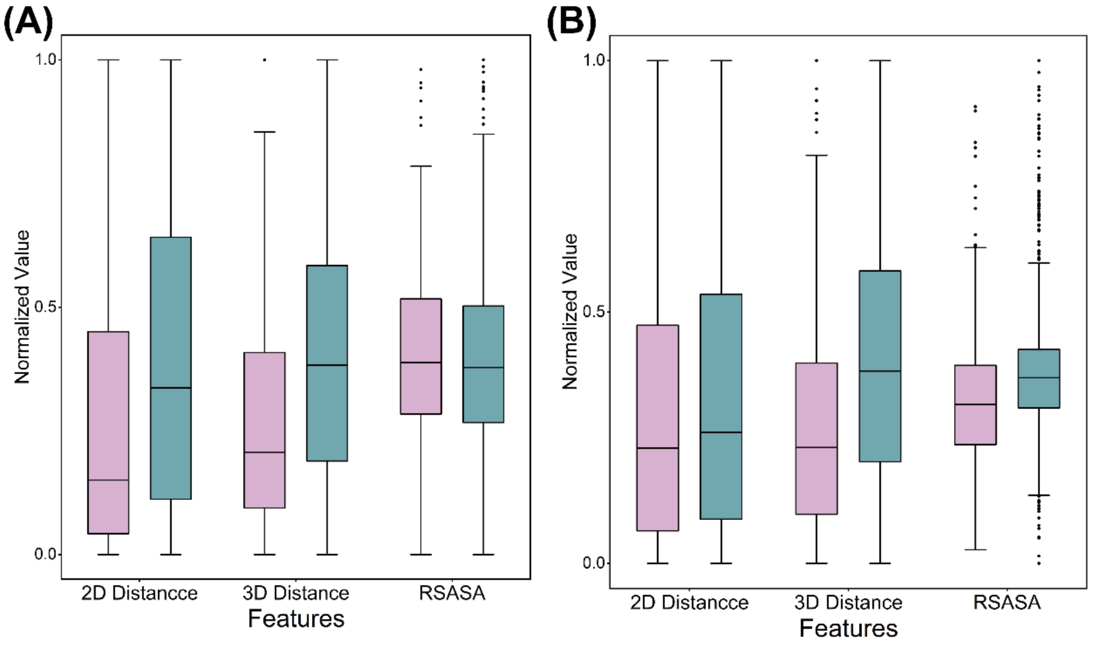

Figure S11. Feature distribution in 2D distance, 3D distance, and RSASA of binding and non-binding sites in (A) TE18 and (B) JL10 test sets. Binding sites are labeled in purple, and non-binding sites are labeled in green.

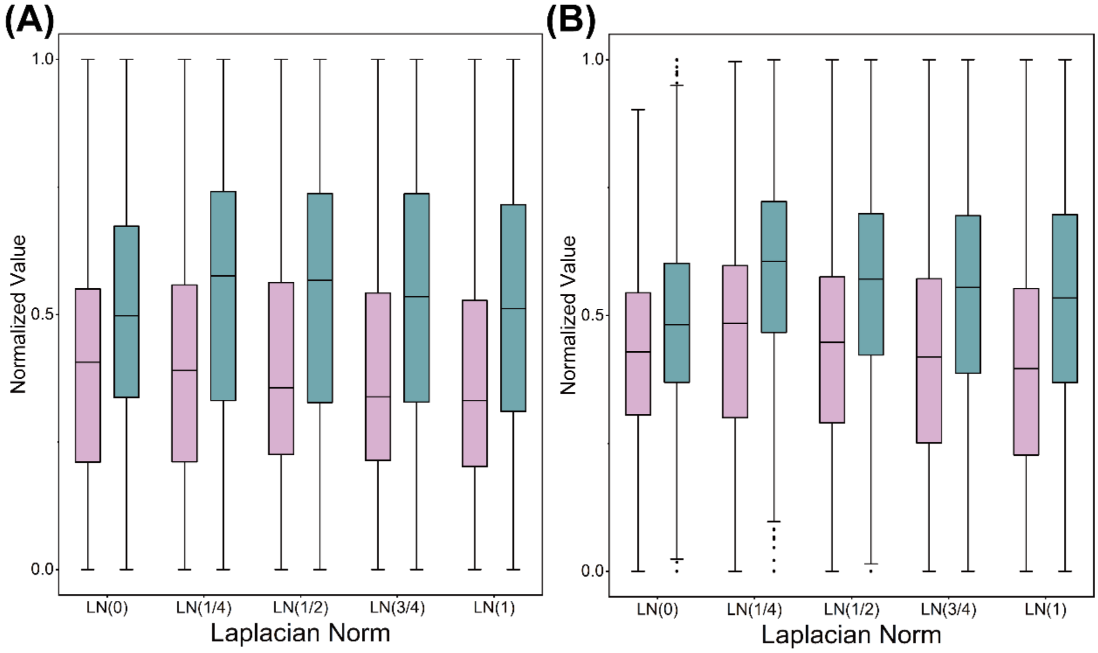

Figure S12. Feature distribution in Laplacian Norm (LN) of binding and non-binding sites in (A) TE18 and (B) JL10 test sets. Binding sites are labeled in purple, and non-binding sites are labeled in green.

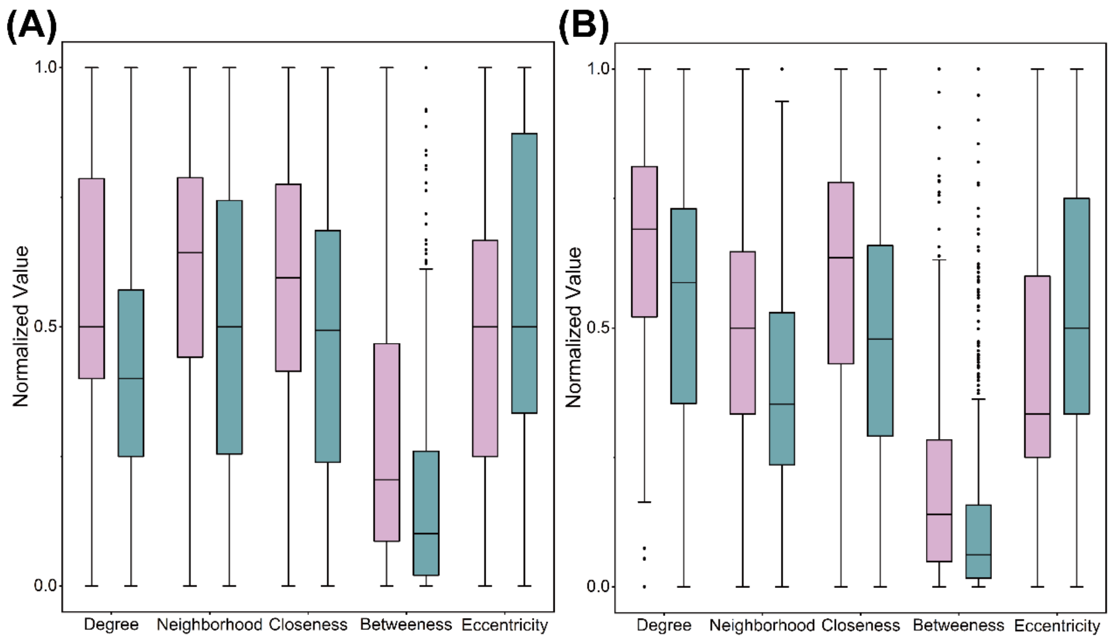

Figure S13. Feature distribution in network features (degree, neighborhood connectivity, closeness, betweenness, and eccentricity) of binding and non-binding sites in (A) TE18 and (B) JL10 test sets. Binding sites are labeled in purple, and non-binding sites are labeled in green.

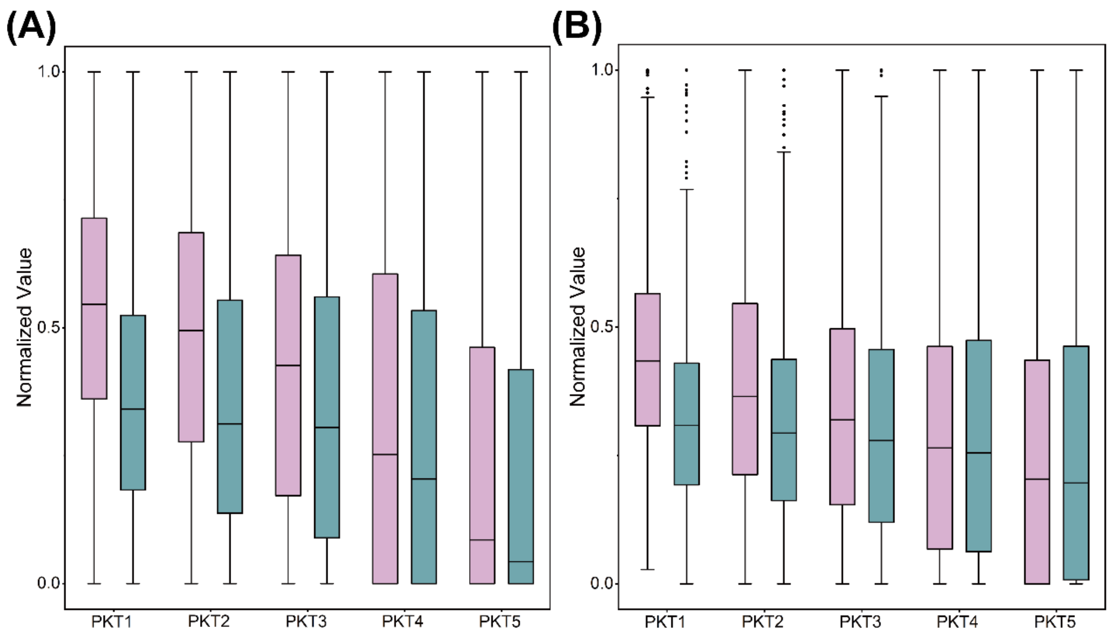

Figure S14. Feature distribution in pocketness of binding and non-binding sites in (A) TE18 and (B) JL10 test sets. Binding sites are labeled in purple, and non-binding sites are labeled in green.

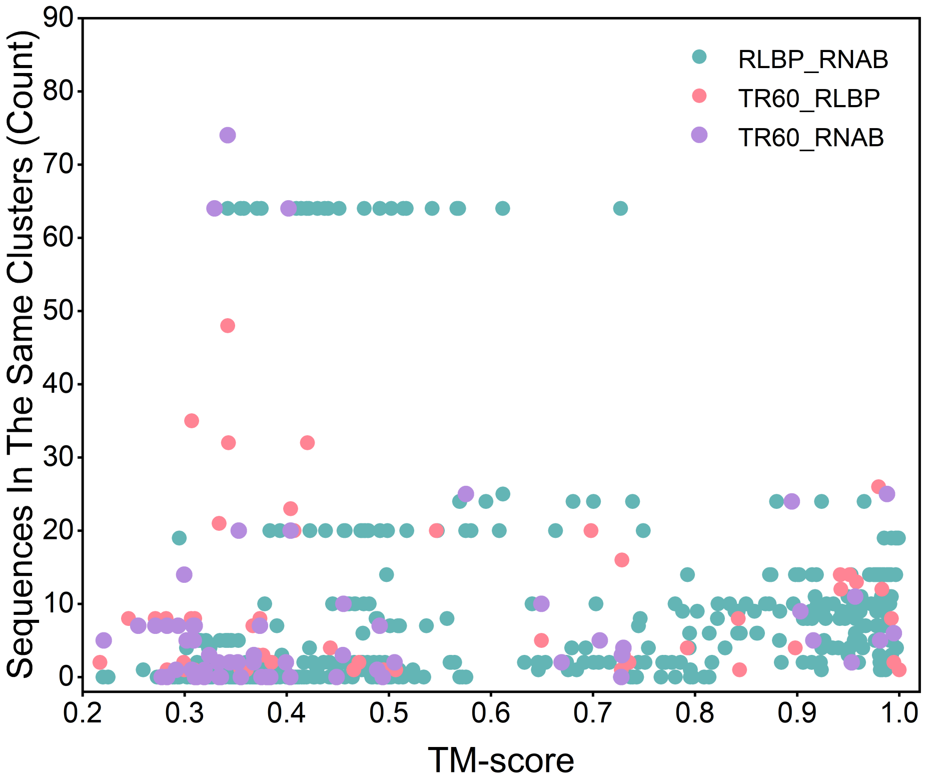

Figure S15. Sequence and structure similarity of three training sets (TR60, trainRLBP, and training set of RNABind). RLBP represents trainRLBP, and RNAB stands for the training set of RNABind. Each dot depicts an RNA in the dataset; the x-coordinate shows the maximum TM-score between the query RNA and any structure in the other dataset, while the y-coordinate indicates the number of RNA chains in the other dataset that fall into the same cluster as the query RNA.
